# A whole-body FDG-PET/CT Dataset with manually annotated Tumor Lesions

**DOI:** 10.1038/s41597-022-01718-3

**Published:** 2022-10-04

**Authors:** Sergios Gatidis, Tobias Hepp, Marcel Früh, Christian La Fougère, Konstantin Nikolaou, Christina Pfannenberg, Bernhard Schölkopf, Thomas Küstner, Clemens Cyran, Daniel Rubin

**Affiliations:** 1 Max-Planck-Institute for Intelligent Systems, Empirical Inference Department, Tuebingen, 72076 Germany; 2 University Hospital Tübingen, Department of Radiology, Tübingen, 72076 Germany; 3 University Hospital Tübingen, Department of Nuclear Medicine and Clinical Molecular Imaging, Tübingen, 72076 Germany; 4 Cluster of Excellence iFIT (EXC 2180) “Image-Guided and Functionally Instructed Tumor Therapies”, Tübingen, 72076 Germany; 5 German Cancer Consortium (DKTK), German Cancer Research Center (DKFZ) Partner Site Tübingen, Tübingen, 72076 Germany; 6 Hospital of the Ludwig-Maximilians-University, Department of Radiology, Munich, 81377 Germany; 7 Stanford University, School of Medicine, Department of Biomedical Data Science, Stanford, 94305 USA

**Keywords:** Diagnostic markers, Whole body imaging, Cancer imaging

## Abstract

We describe a publicly available dataset of annotated Positron Emission Tomography/Computed Tomography (PET/CT) studies. 1014 whole body Fluorodeoxyglucose (FDG)-PET/CT datasets (501 studies of patients with malignant lymphoma, melanoma and non small cell lung cancer (NSCLC) and 513 studies without PET-positive malignant lesions (negative controls)) acquired between 2014 and 2018 were included. All examinations were acquired on a single, state-of-the-art PET/CT scanner. The imaging protocol consisted of a whole-body FDG-PET acquisition and a corresponding diagnostic CT scan. All FDG-avid lesions identified as malignant based on the clinical PET/CT report were manually segmented on PET images in a slice-per-slice (3D) manner. We provide the anonymized original DICOM files of all studies as well as the corresponding DICOM segmentation masks. In addition, we provide scripts for image processing and conversion to different file formats (NIfTI, mha, hdf5). Primary diagnosis, age and sex are provided as non-imaging information. We demonstrate how this dataset can be used for deep learning-based automated analysis of PET/CT data and provide the trained deep learning model.

## Abstract (summary)

**Table:** 

Measurement(s)	tumor lesions
Technology Type(s)	PET/CT
Sample Characteristic - Organism	Homo sapiens

## Background & Summary

Integrated Positron Emission Tomography/Computed Tomography (PET/CT) has been established as a central diagnostic imaging modality for several mostly oncological indications over the past two decades. The unique strength of this hybrid imaging modality lies in its capability to provide both, highly resolved anatomical information by CT as well as functional and molecular information by PET. With growing numbers of performed examinations, the emergence of novel PET tracers and the increasing clinical demand for quantitative analysis and reporting of PET/CT studies is becoming increasingly complex and time consuming. To overcome this challenge, the implementation of machine learning algorithms for faster, more objective and quantitative medical image analysis has been proposed also for the analysis of PET/CT data. First methodological studies have demonstrated the feasibility of using deep learning frameworks for the detection and segmentation of metabolically active lesions in whole body Fluorodeoxyglucose (FDG)-PET/CT of patients with lung cancer, lymphoma and melanoma^1–4^. Despite these encouraging results, deep learning-based analysis of PET/CT data is still not established in routine clinical settings. Thus, automated medical image analysis, specifically of PET/CT images is an ongoing field of research that requires methodological advances to become clinically applicable. In contrast to the more widely used imaging modalities CT and MRI however, only few datasets of PET/CT studies are publicly accessible to clinical and machine learning scientists who work on automated PET/CT analysis. Even fewer datasets contain image-level ground truth labels to be used for machine learning research^5,6^. This is likely a major obstacle for innovation and clinical translation in this field. Examples of related areas, such as analysis of dermoscopy^7^ or retinal images^8^, show that the existence of publicly available labeled datasets can serve as a catalyst for method development and validation. The purpose of this project is thus to provide an annotated, publicly available dataset of PET/CT images that enables technical and clinical research in the area of machine learning-based analysis of PET/CT studies and to demonstrate a use case of deep learning-based automated segmentation of tumor lesions. To this end, we composed a dataset of 1,014 oncologic whole-body FDG-PET/CT examinations of patients with lymphoma, lung cancer and malignant melanoma, as well as negative controls together with voxel-wise manual labels of metabolically active tumor lesions. The provided data can be used by researchers of different backgrounds for the development and evaluation of machine learning methods for PET/CT analysis as well as for clinical research regarding the included tumor entities.

## Methods

### Data collection

Publication of anonymized data was approved by the institutional ethics committee of the Medical Faculty of the University of Tübingen as well as the institutional data security and privacy review board. Data from 1,014 whole-body FDG-PET/CT examinations of 900 patients acquired between 2014 and 2018 as part of a prospective registry study^9^ were included in this dataset. Of these 1,014 examinations, 501 are positive samples, meaning they contain at least one FDG-avid tumor lesion and 513 are negative samples, meaning they do not contain FDG-avid tumor lesions. Negative samples stem from patients who were examined by PET/CT with a clinical indication (e.g. follow-up after tumor resection) but did not show any findings of metabolically active malignant disease. The selection criteria for positive samples were: age >18 years, histologically confirmed diagnosis of lung cancer, lymphoma or malignant melanoma, and presence of at least one FDG-avid tumor lesion according to the final clinical report. The selection criteria for negative samples were: age >18 years, no detectable FDG-avid tumor lesion according to the clinical radiology report. Of the 501 positive studies, 168 were acquired in patients with lung cancer, 145 in patients with lymphoma and 188 in patients with melanoma. Patient characteristics are summarized in Table 1.

**Table 1:** Patient characteristics across the dataset subcategories.

diagnosis	patient sex	n/o studies	age [mean SD]
Melanoma	female	77	65.0 ± 12.8
	male	111	65.7 ± 13.7
Lymphoma	female	69	45.1 ± 19.7
	male	76	47.3 ± 17.9
Lung Cancer	female	65	64.2 ± 8.7
	male	103	67.0 ± 9.0
Negative	female	233	59.1 ± 14.7
	male	280	58.7 ± 15.1
All	female	444	58.5 ± 16.1
	male	570	60.1 ± 15.9

### PET/CT Acquisition

All PET/CT examinations were performed at the University Hospital Tübingen according to a standardized acquisition protocol on a single clinical scanner (Siemens Biograph mCT, Siemens Healthineers, Knoxville, USA) following international guidelines for oncologic PET/CT examinations (Boellaard *et al*. FDG PET/CT: EANM procedure guidelines for tumour imaging: version 2.0)^10^.

Diagnostic whole-body CT was acquired in expiration with arms elevated according to a standardized protocol using the following scan parameters: reference tube current exposure time product, 200 mAs with automated exposure control (CareDose); tube voltage, 120 kV. CT examinations were performed with weight-adapted 90–120 ml intravenous CT contrast agent in a portal-venous phase (Ultravist 370, Bayer Healthcare) or without contrast agent (in case of existing contraindications). CT data were reconstructed in transverse orientation with a slice thickness between 2.0 mm and 3.0 mm with an in-plane voxel edge length between 0.7 and 1.0 mm.

**Fig. 1:**
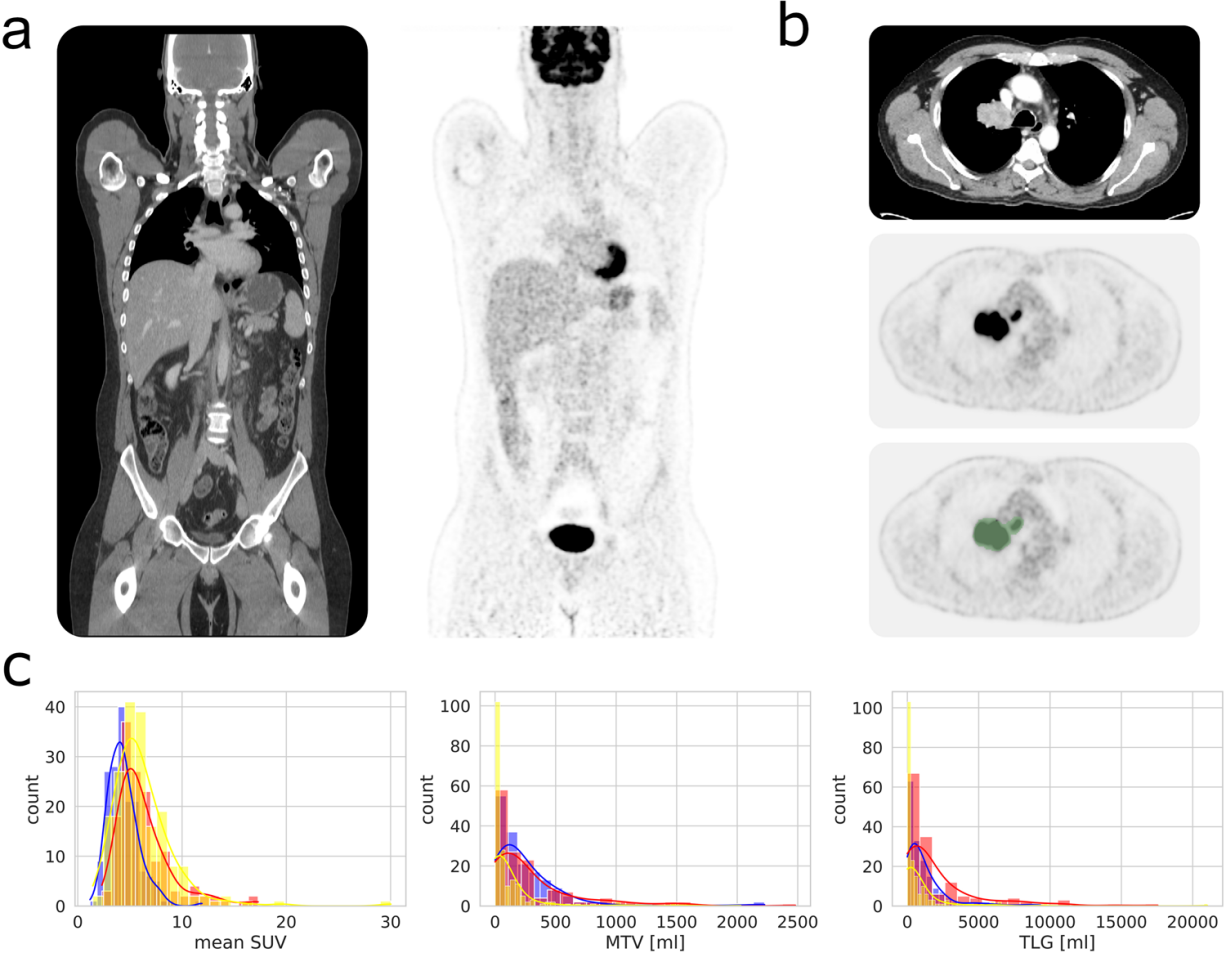
Dataset properties. (**a**) Coronal views of CT (left) and FDG-PET (right) image volumes without pathologic findings. (**b**) Example of manual tumor segmentation (bottom image, green area) of a lung cancer mass; top: CT, middle: FDG-PET (**c**) Distribution of mean SUV, MTV and TLG of studies in patients with lung cancer (blue), lymphoma (red) and melanoma (yellow).

^18^F-FDG was injected intravenously after at least 6 hours of fasting. PET acquisition was initiated 60 minutes after injection of a weight-adapted dose of approximately 300 MBq 18F-FDG (mean: 314.7 MBq, SD: 22.1 MBq, range: [150, 432] MBq). For the purpose of weight adaptation, target FDG injection acitivities were 250–300 MBq/300–350 MBq/350–400 MBq for patients with a body weight below 60 kg/between 60 and 100 kg/above 100 kg respectively. PET was acquired over four to eight bed positions (usually from the skull base to the mid-thigh level) and reconstructed using a 3D-ordered subset expectation maximization algorithm (two iterations, 21 subsets, Gaussian filter 2.0 mm, matrix size 400 × 400, slice thickness 3.0 mm, voxel size of 2.04 × 2.04 × 3 mm^3^). PET acquisition time was 2 min per bed position. Example PET/CT images are displayed in Fig. 1a.

### Data labeling and processing

All examinations were assessed by a radiologist and nuclear medicine specialist in a clinical setting. Based on the report of this clinical assessment, all FDG-avid tumor lesions (primary tumor if present and metastases if present) were segmented by an experienced radiologist (S.G., 10 years of experience in hybrid imaging) using dedicated software (NORA image analysis platform, University of Freiburg, Germany). In case of uncertainty regarding lesion definition, the specific PET/CT studies were reviewed in consensus with the radiologist and nuclear medicine physician who prepared the initial clinical report. To this end CT and corresponding PET volumes were displayed side by side or as an overlay and tumor lesions showing elevated FDG-uptake (visually above blood-pool levels) were segmented in a slice-per-slice manner resulting in 3D binary segmentation masks. An example slice of a segmented tumor lesion is shown in Fig. 1b. DICOM data of PET/CT volumes and corresponding segmentation masks were anonymized upon data upload to The Cancer Imaging Archive^11^ using the CTP DICOM anonymizer tool.

### Data properties

Of the 1014 studies (900 unique patients) included in this dataset, one study was included of 819 patients, two studies were included of 59 patients, 3 studies of 14 patients, 4 studies of 4 patients and 5 studies of 3 patients. The mean coverage (scan range) of the PET volumes in the longitudinal direction over all datasets was 1050.7 mm (SD: 306.7 mm, min: 600 mm, max: 1983 mm). The three included tumor entities showed similar distributions with respect to metabolic tumor volume (MTV), mean SUV of tumor lesions and total lesion glycolysis (TLG) (Fig. 1c). Overall, in non-negative studies, MTV, mean SUV and TLG amounted to (mean ± SD) 219.9 ± 342.7 ml, 5.6 ± 2.7 and 1330 ± 2296 ml, respectively. For lung cancer studies, these values were 263.6 ± 345.1 ml, 4.4 ± 1.5 and 1234 ± 1622 ml. For lymphoma studies these values 297.5 ± 393.1 ml, 6.3 ± 2.7 and 2042 ± 2941.4 ml. For melanoma studies these values were 121.2 ± 269.4 ml, 6.2 ± 3.1 and 867.3 ± 2113.8 ml.

## Data Records

This dataset can be accessed on The Cancer Imaging Archive (TCIA) under the collection name “FDG-PET-CT-Lesions”^12^.

### DICOM data

**Fig. 2:**
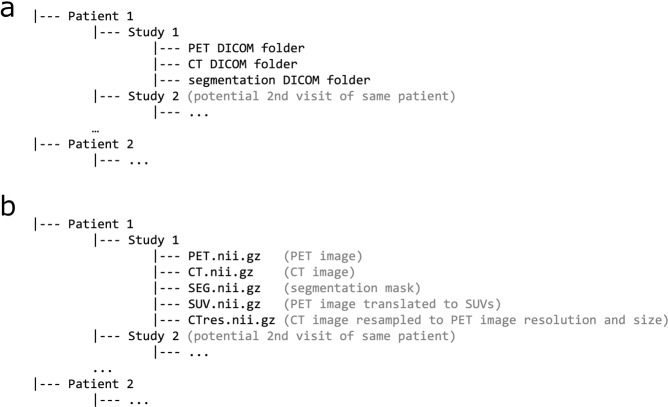
Dataset structure. Patients are identified by a unique, anonymized ID and all studies of a single patient are stored under the respective patient path. (**a**) DICOM data: Each study folder contains three subfolders with DICOM files of the PET volume, the CT volume and the segmentation mask. (**b**) NIfTI data: Using the provided conversion script, DICOM data can be converted to NIfTI files. In addition to NIfTI files of the PET volume (PET.nii.gz), the CT volume (CT.nii.gz) and the segmentation mask (SEG.nii.gz), this script generates NIfTI volumes of the PET image in SUV units (SUV.nii.gz) and a CT volume resample to the PET resolution and shape (CTres.nii.gz).

Each individual PET/CT dataset consists of three image series stored in the DICOM format: a whole-body CT volume stored as a DICOM image series, a corresponding whole-body FDG-PET volume stored as a DICOM image series and a binary segmentation volume stored in the DICOM segmentation object format. The entire DICOM dataset consists of 1,014 image studies, 3,042 image series and a total of 916,957 single DICOM files (total size of approximately 419 GB). The directory structure of the DICOM dataset is depicted in Fig. 2a. Patients are identified uniquely by their anonymized patient ID.

### Conversion to other image formats

To facilitate data usage, we provide Python scripts that allow conversion of DICOM data to other medical image formats (NIfTI and mha) as well as the hdf5 format. (https://github.com/lab-midas/TCIA processing). In addition to file conversion, these scripts generate processed image volumes: a CT volume resampled to the PET volume size and resolution as well as a PET volume with voxel values converted to standardized uptake values (SUV). The data structure of the generated NIfTI files is represented in Fig. 2b. Data in the other formats (mha and hdf5) are generated accordingly.

### Metadata

In addition to imaging data, a metadata file in Comma-separated Values (csv) format is provided containing information on study class (lung cancer, melanoma, lymphoma or negative), patient age (in years) and patient sex. In addition, the DICOM header data include information about patient body weight, injected activity and whether CT was contrast-enhanced (in case of non-enhanced CT, the CT series description includes the key word “nativ”).

## Technical Validation: Deep Learning-based Lesion Segmentation

**Fig. 3:**
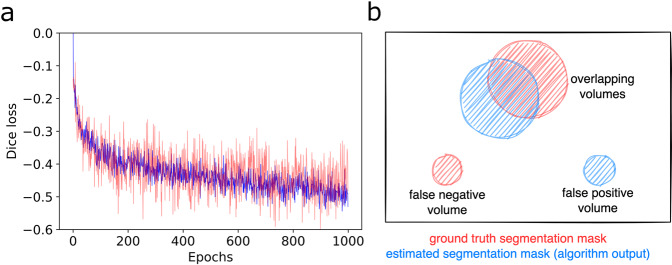
Training and evaluation. (**a**) Representative loss curve on training data (blue) and validation data (red) from one fold of a 5-fold cross validation. (**b**) Schematic visualization of the proposed evaluation metrics false positive and false negative volumes (in addition to the Dice score).

In order to provide a use case scenario for the provided dataset we trained and evaluated a deep learning model for automated PET lesion segmentation. To this end, we used a standardized and publicly available deep learning framework for medical image segmentation (nnUNet^13^). This framework is based on a 3D U-Net architecture and provides an automated adaptive image processing pipeline. PET volumes converted to SUV units (SUV.nii.gz, Fig. 2b) and corresponding re-sampled CT volumes (CTres.nii.gz, Fig. 2b) were used as model inputs. Training with 5-fold cross validation was performed using the pre-configured model parameters with maximum number of epochs set to 1,000 and an initial learning rate of 1e-4 in a dedicated GPU (NVIDIA A5000). Typical loss and validation curves of a single validation step are depicted in Fig. 3a. For validation of algorithm performance, three metrics were used: Dice score, false positive volume and false negative volume (Fig. 3b). False positive volume was defined as the volume of false positive connected components in the predicted segmentation mask that do not overlap with tumor regions in the ground truth segmentation mask. This can be e.g. areas of physiological FDG-uptake (e.g. brain, heart, kidneys) that are erroneously classified as tumor. False negative volume was defined as the volume of connected components in the ground truth segmentation mask (=tumor lesions) that do not overlap with the estimated segmentation mask. These are tumor lesions that are entirely missed by the segmentation algorithm. In case of negative examples without present tumor lesions in the ground truth segmentation, only false positive volume was applicable as a metric.

We introduce these additional metrics (false positive and false negative volumes) due to the specific requirements of automated lesion segmentation. The specific challenge in automated segmentation of FDG-avid lesions in PET is to avoid false-positive segmentation of anatomical structures that have physiologically high FDG-uptake (e.g. brain, kidney, heart, etc.) while capturing all - even small - tumor lesions. The Dice score alone does not differentiate between false positive or negative segmentation within a correctly detected lesion (e.g. along its borders) and false positive or negative segmentations unconnected to detected lesions (i.e. false positive segmentation of healthy tissue or entirely missed lesions).

**Fig. 4:**
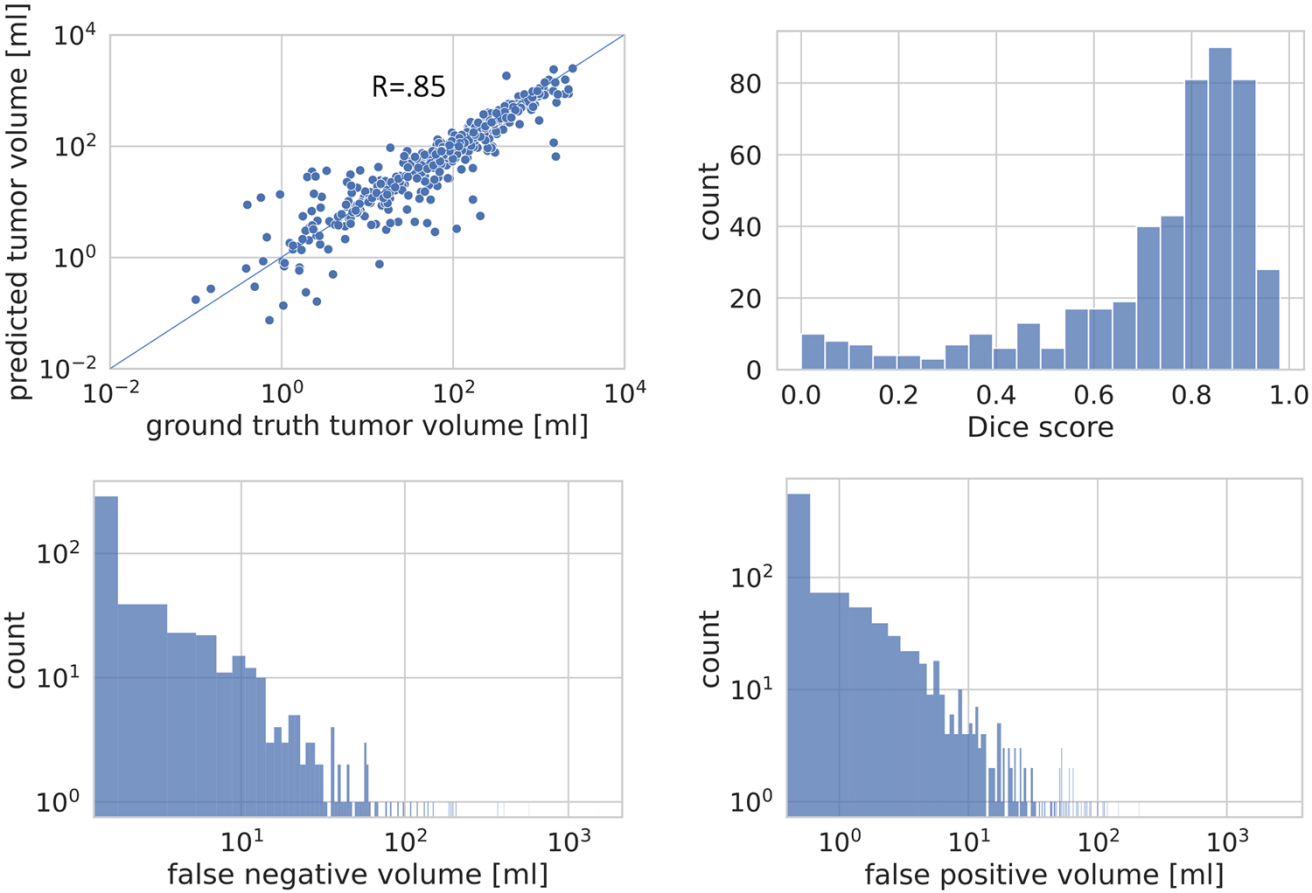
Quantitative evaluation of automated lesion segmentation. Top left: Correlation of automatically predicted tumor volume with ground truth tumor volumes from manual segmentation in positive studies. Top right: Distribution of Dice coefficients for automated versus manual tumor segmentation in positive studies. Bottom left: Distribution of false negative volumes over all positive studies. Bottom right: Distribution of false positive volumes over all studies.

**Fig. 5:**
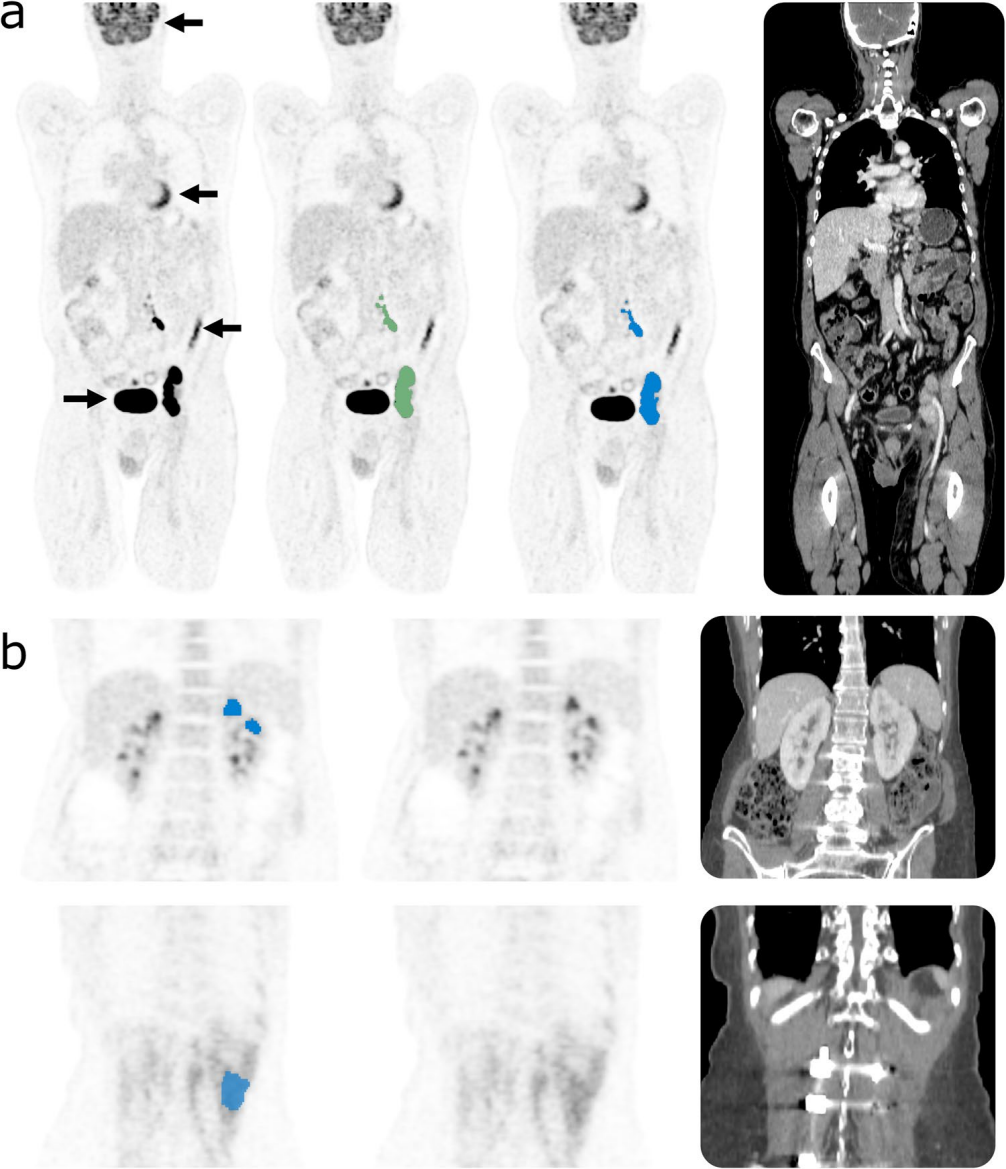
Examples of automated lesion segmentation. (**a**) Example showing excellent agreement between manual (green) and automated (blue) tumor segmentation in a patient with lymphoma. Black arrows point to physiological FDG-uptake in the brain, heart, bowel and urinary bladder (from top to bottom) that was correctly not segmented. (**b**) Example of false positive segmentation of physiological structures with elevated FDG-uptake. Top: False positive partial segmentation of the left kidney. Bottom: False positive partial segmentation of back muscles.

Overall, automated lesion segmentation using the described deep learning model showed good agreement with manual ground truth segmentation (Fig. 4). On datasets containing lesions, a high correlation of MTVs was observed between automated and manual segmentation (*r* = 0.85). The mean Dice score of automated compared to manual lesion segmentation was 0.73 (±0.23) on positive datasets. Mean false positive/false negative volumes were 8.1 (±81.4) ml/15.1 (±80.3) ml respectively. Quantitative algorithm performance results on validation data (5-fold cross validation) are summarized in Fig. 4. Figure 5 provides qualitative examples for automated segmentation results.

This presented use case scenario demonstrates how this dataset can be used for the development and validation of algorithms for analysis of PET/CT data. We observed overall high automated segmentation performance that is comparable to previous studies focusing on specific disease entities^4,14^. In combination with methodological advances in the fields of machine learning and computer vision, this dataset can thus contribute to the development of increasingly accurate, robust and clinically useful algorithms for PET/CT analysis.Beyond automated lesion segmentation, this dataset bears the potential to be used for further tasks such as automated organ segmentation or automated lesion tracking. This would require further annotations which can be integrated with relatively low additional effort. For example, the recently published MOOSE framework^15^ for automated organ segmentation on PET/CT data can be directly applied to this dataset providing e.g. information about lesions localization.

## Usage Notes

For the purpose of visualization, image data can be loaded using freely available medical image data viewers such as the Medical Imaging Interaction Toolkit (https://www.mitk.org/) or 3D Slicer (https://www.slicer.org/). For the purpose of computational data analysis e.g. in Python, 3D image volumes can be read using freely available software such as pydicom (https://pydicom.github.io/) or nibabel (https://nipy.org/packages/nibabel/index.html). The data presented in this manuscript is part of the MICCAI autoPET challenge 2022 (https://autopet.grand-challenge.org/).

## Data Availability

We provide the code of the data conversion and processing pipeline under https://github.com/lab-midas/TCIA processing. The trained PET/CT lesion segmentation model is publicly available under https://github.com/lab-midas/autoPET/tree/master/.
